# Festival Dinner

**Published:** 1904-06-04

**Authors:** 


### FESTIVAL DINNER.

### KING'S COLLEGE HOSPITAL.

H.R.H. the Duke of Connaught presided over the festival dinner of King's College Hospital, which was held at the Hôtel Cecil on Monday, May 30th.

In proposing the toast of "The King," His Royal Highness said they must remember that he was the founder of the King Edward's Hospital Fund, from the working of which they all hoped for so much. (Applause.) In responding to the toast of "The Queen and the rest of the Royal Family," he said that all knew the great interest Queen Alexandra took in all hospitals whether civil, military, or naval. (Hear, hear.) Her Majesty had tried in every way to replace our late Queen in everything connected with the medical welfare of all parts of the Empire. (Applause.) The Prince and Princess of Wales had done all they could to support their Majesties in the interest they took in the welfare of the hospitals—(applause)—and for himself, his taking the chair that evening, showed in a small way the interest he took in hospitals. (Hear, hear.) In proposing the toast of the evening, "Success to King's College Hospital," he said he did so with confidence and with pleasure. With confidence because from all he had heard he thought that King's College Hospital was one which appealed very strongly to a very large number of gentlemen in the metropolis.

The hospital had the distinction of having had Lord Lister very closely connected with them. What Lord Lister had done was of the greatest benefit to humanity—he had done much to alleviate the suffering and to aid the recovery of the sick. (Applause.) He had only heard that evening a very interesting fact, viz., that when Lord Lister first came to the hospital the deaths resulting from operations used to be one in three—when his system had been adopted the number had fallen to one in three hundred. (Applause.) Referring to the approaching removal of the hospital to Camberwell, his Royal Highness said that the present hospital had proved inadequate for the increasing medical necessities of the district, the buildings were too small for the patients, for the medical staff, and for all that now went to make up a modern hospital. It had been found necessary to increase it in every department. These increases were impossible on the present site, and it had been wisely decided to remove the hospital to another part of London. An admirable site had been secured, and he trusted that when the hospital was moved there it would be found that its work would be advantageously extended.

Lord Methuen, the Chairman of the Hospital Committee, responded. He said that since he had accepted that position what surprised him most was the splendid way in which people, who he knew experienced the greatest difficulty in giving their mite, had put their hands in their pockets and had come forward to the assistance of the hospital in the present emergency. (Applause.) At the present time the hospital was taking a grave step, the gravest it had taken during its existence, and they looked to its supporters to give them that help which he felt sure they would receive from the inhabitants of the Metropolis. (Applause.)

After the toasts of "The Church" (proposed by Mr. Justice Warrington, replied to by the Archbishop of Canterbury), and "The Army, Navy, and Reserve Forces" (proposed by Sir E. C. K. Ollivant, and replied to by Admiral the Hon. Sir E. R. Fremantle and General Sir F. Forestier-Walker), the Rev. A. C. Headlam proposed the "Medical and Nursing Staff of the Hospital." In the nursing staff, he said, they had one which was unequalled in the kingdom (hear, hear)—while the head staff included some of the most distinguished medical men in the country; men who were closely connected with the advance of medical science. (Applause.)

Dr. Ferrier, whose rising was greeted with loud applause, in responding, said the subject which at the present time they had on their minds was not so much the existing but the future hospital. As to the existing hospital, he thought they might say that the patients had always been well treated and as well looked after as in any hospital in the Kingdom, but they only looked at the present hospital as a stage in the evolution of something better, something more perfect. The medical staff were expected to do something more than apply medical science, they were expected to advance it, and to leave it, if possible, in a better position than they found it; but in their efforts in that direction they were hampered by want of room, by want of light, and their work had to be carried on under conditions which were far from being good. Therefore they were all anxious to see a new King's College Hospital replete with every comfort for the patients, and furnished with every appliance for the study and treatment of disease which latter-day science could provide. (Applause.) For this object the medical staff were co-operating in entire harmony with the board of management, and they were greatly indebted to his Royal Highness for the encouragement he had given them in presiding on that occasion. (Applause.)

The toast of "the Chairman" concluded the proceedings. His Royal Highness, in responding, said he had had the great honour of being asked to become the President of the hospital, and he had the greatest pleasure in acceding to that request, succeeding, as he did, a very near relative who had recently passed away.

The Treasurer (Mr. Charles Awdry) announced that the amount collected as the result of the festival was £2,160.

